# Hobnail hemangioma – Dermoscopic variability of an uncommon acquired lymphatic malformation

**DOI:** 10.1016/j.jdcr.2026.03.065

**Published:** 2026-04-13

**Authors:** Katja Grossschaedl, Rainer Hofmann-Wellenhof, Teresa Kränke

**Affiliations:** Department of Dermatology and Venereology, Medical University of Graz, Graz, Austria

**Keywords:** hobnail hemangioma, lymphatic malformation, targetoid hemosiderotic hemangioma, vascular malformation

## Case presentation

**Fig 1:**
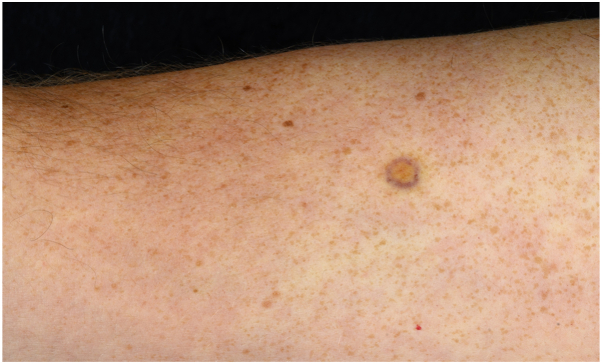
Clinical image of the patient’s right upper arm, showing a clearly circumscribed, central yellowish-brown nodule measuring 8 mm with a livid reddish-violet peripheral rim (A).

A 41-year old male presented to our emergency dermatology department after noticing a painless yellowish-brown nodule with a livid peripheral rim on his right upper arm. No history of trauma was reported. The clinical examination revealed a solitary, well-circumscribed, firm nodule measuring 8 mm in diameter (Fig 1).

## Dermoscopic appearance

**Fig 2:**
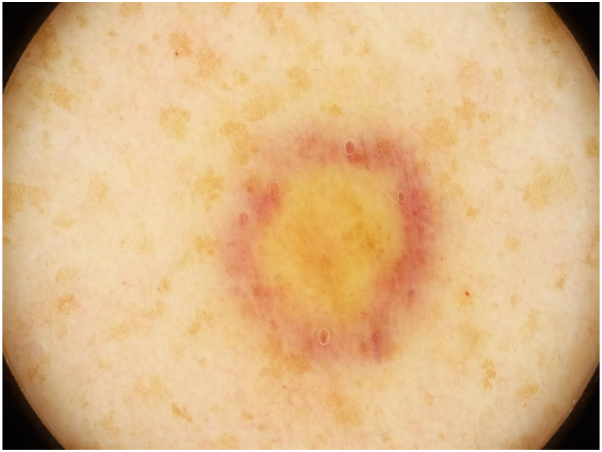
Dermoscopic image exhibiting the nodule’s yellowish-brown homogenous center, network of brown pigmented tissue, and the surrounding livid reddish-violet peripheral rim.

A closer examination of the lesion indicated that it had a central, nearly homogenous yellowish-brown area with a reddish-violet peripheral rim. Additionally observed brown areas with homogeneous yellow centers were believed to correspond to the freckles/lentigines seen on the patient’s arm. No lacunae or vessels were detected (Fig 2).

## Histopathologic diagnosis

Histopathologic examination revealed the presence of dilated vessels in the dermis, with erythrocyte extravasation and hemosiderin-laden macrophages. Most of the vessels stained positive for Podoplanin, suggesting a lymphatic component. No evidence of malignancy or a melanocytic tumor was found. The lesion was diagnosed as a hobnail hemangioma.

Key message

Hobnail hemangioma, also known as targetoid hemosiderotic hemangiomais, is a rare, acquired benign malformation predominantly affecting adults, which is slightly more common in men. Although these lesions were previously considered to be proliferative vascular lesions, they are now considered to be evidence of a hemosiderotic lymphatic malformation. Variable clinical and dermoscopic findings may indicate the presence of hobnail hemangioma, depending on the stage of cyclical change.^1^ Differential diagnoses include infantile hemangiomas, melanocytic nevi, melanoma, Kaposi sarcoma, hemangioma, angiokeratoma, and dermatofibroma.^2^ In accordance with its typical clinical presentation, the most frequently observed dermoscopic pattern consisted of central red to violet lacunae, each of which had a reddish-violet homogeneous peripheral rim.^2^ This peripheral rim may offer an useful diagnostic clue; however, this observation was made at a single time point, and these lesions are well known to undergo dynamic changes. The most frequently reported evolutive features include the gradual loss of the central lacunae, alterations in the coloration, and regression of vascular structures, among others. Our case highlights the diagnostic challenges presented by hobnail hemangiomas and underscores the importance of performing an early histopathologic evaluation of ambiguous skin lesions.

## Conflicts of interest

None disclosed.

## References

[1] Bui T., Rezac L.M., Noble C.A., Velasquez-Evers A.R., Brodell R.T. (2025). Targetoid hemosiderotic hemangioma: a review article. Semin Diagn Pathol.

[2] Zaballos P., Llambrich A., Del Pozo L.J. (2015). Dermoscopy of targetoid hemosiderotic hemangioma: a morphological study of 35 cases. Dermatology.

